# Intraosseous Pneumatocysts of the Scapula Mimicking Bone Tumors: A Report of Two Rare Cases Along with Elucidation of Their Etiology

**DOI:** 10.3390/diseases13060170

**Published:** 2025-05-27

**Authors:** Jiro Ichikawa, Masanori Wako, Tomonori Kawasaki, Satoshi Ochiai, Tetsuo Hagino, Naofumi Taniguchi, Kouhei Mitsui, Kojiro Onohara

**Affiliations:** 1 Department of Orthopaedic Surgery, Interdisciplinary Graduate School of Medicine, University of Yamanashi, Chuo, Kofu 409-3898, Japan; wako@yamanashi.ac.jp (M.W.); naofumit@yamanashi.ac.jp (N.T.); koumitsui@yamanashi.ac.jp (K.M.); 2 Department of Pathology, Saitama Medical University International Medical Center, Hidaka 350-1298, Japan; tomo.kawasaki.14@gmail.com; 3 Department of Orthopaedic Surgery, National Hospital Organization (NHO) Kofu National Hospital, Kofu 400-8533, Japan; hxcmk230@ybb.ne.jp (S.O.); tmhagino@amber.plala.or.jp (T.H.); 4 Department of Radiology, Interdisciplinary Graduate School of Medicine, University of Yamanashi, Chuo, Kofu 409-3898, Japan; konohara@yamanashi.ac.jp

**Keywords:** pneumatocyst, magnetic resonance imaging, computed tomography, differential diagnosis

## Abstract

Background/Objectives: Pneumatocysts, characterized by gas-filled cavities, are commonly found in the spine and pelvis but are rarely observed in the scapula. In this report, we describe two rare cases of scapular pneumatocysts mimicking bone tumors and exhibiting different image findings. Case Report: Case 1. A 47-year-old man who presented with neck pain underwent radiography, followed by magnetic resonance imaging (MRI). MRI showed heterogeneity with low and high signals on fat-suppressed T2-weighted images, suggestive of enchondroma or fibrous dysplasia (FD). However, preoperative computed tomography (CT) revealed gas-filled cavities within the tumor, in continuity with the shoulder joint, confirming the diagnosis of a pneumatocyst. Case 2. A 58-year-old woman who presented with neck pain underwent similar examinations to Case 1. MRI showed homogeneity with high signals on fat-suppressed T2-weighted images, leading to a suspicion of solitary bone cysts and FD. Preoperative CT revealed gas-filled cavities within the tumor, but no continuity with the joint, leading to the diagnosis of a pneumatocyst. While the exact etiology of pneumatocysts remains unclear, two potential causes are as follows: (i) gas migration from the joint to the bone, and (ii) gas replacement in cystic tumors. Thus, CT is particularly valuable in confirming the presence of gas-filled cavities and aiding in diagnosis. Conclusions: This report highlights two extremely rare cases of scapular pneumatocysts, reflecting two potential etiologies. The utility of CT in the diagnosis of pneumatocyst has been clarified.

## 1. Introduction

Pneumatocysts are defined as gas-filled bone lesions [1]. They are usually asymptomatic and are discovered incidentally. They are commonly found in the spine and ilium, with less-frequent occurrences in other bones [1,2]. We searched radiology reports from [2000] to [2025] using the keywords “[Pneumatocyst]” and “[Sacapula]”. Two cases with available CT and/or MRI images were identified and included in this study. Two cases of pneumatocysts of the scapula have been reported to date [3,4]. Although gas-fluid levels can occasionally be observed on magnetic resonance imaging (MRI), the differential diagnosis can be challenging at times [3]. On the other hand, computed tomography (CT) can easily demonstrate the presence of gas-filled cavities with CT values ranging from −580 to −950 HU [1]; additionally, continuity between the joint and bone is also noted [3]. While the detailed etiology of a pneumatocyst remains unknown, potential causes include (1) the movement of gas from the joint into the bone, or (2) the spontaneous production of gas in a cystic lesion within the bone.

Herein, we report two rare cases of scapular pneumatocysts, reflecting two potential etiologies. In diagnosing pneumatocysts, CT is actually more helpful than MRI.

## 2. Case Report

We searched radiology reports using the keywords “pneumatocyts” and “scapula”. Two cases with available CT and/or MRI images were identified and included in this study.

### 2.1. Case 1

**Figure 1:**
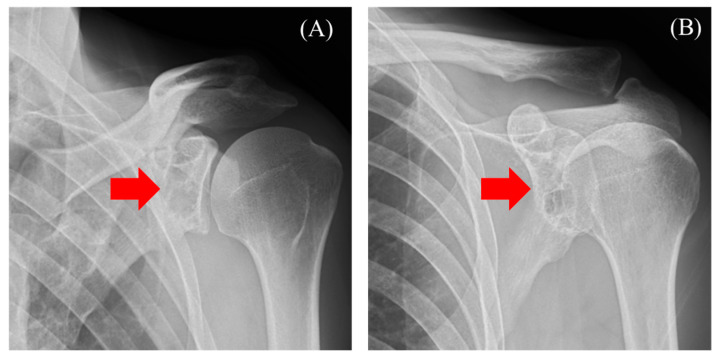
Radiography reveals a radiolucent area with a sclerotic margin ((**A**) red arrow) and ((**B**) red arrow).

The patient was a 47-year-old man who presented with shoulder pain radiating from the left side of the neck to the shoulder, which began two months ago. He had previously consulted a nearby clinic one month prior. The primary pain site was the trapezius muscle, and no tenderness was noted in the shoulder joint. The range of motion of the shoulder joint was intact. Additionally, although the patient experienced numbness in the left palm, there was no sensory dullness, impairment in hand movement, dexterity, or muscle weakness. No history of past illnesses or trauma was recorded. Radiography showed a radiolucent area with a sclerotic margin in the left scapular glenoid, but no narrowing of the joint space or abnormalities in the humeral head were observed (Figure 1A,B, red arrow). Suspecting a benign bone tumor of the scapula, an MRI was performed.

**Figure 2:**
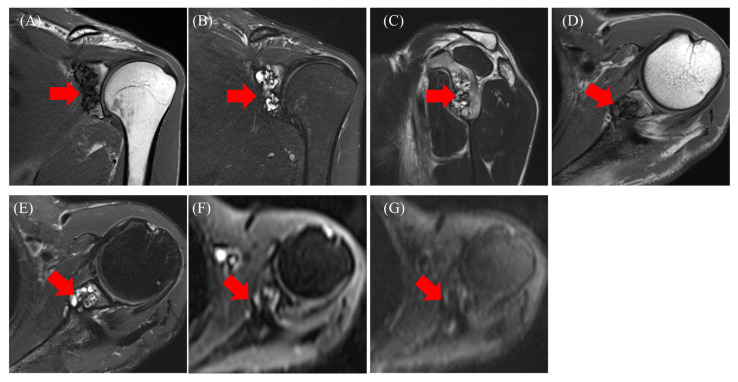
Magnetic resonance imaging: T1-weighted images (**A**,**D**), T2-weighted (**C**), fat-suppressed T2-weighted images (**B**,**E**), diffusion-weighted images b = 0 (**F**), b = 800 (**G**). agnetic resonance imaging: T1-weighted images ((**A**,**D**) red arrow), T2-weighted ((**C**) red arrow), fat-suppressed T2-weighted images ((**B**,**E**) red arrow), diffusion-weighted images b = 0 ((**F**) red arrow), b = 800 ((**G**) red arrow).

**Figure 3:**
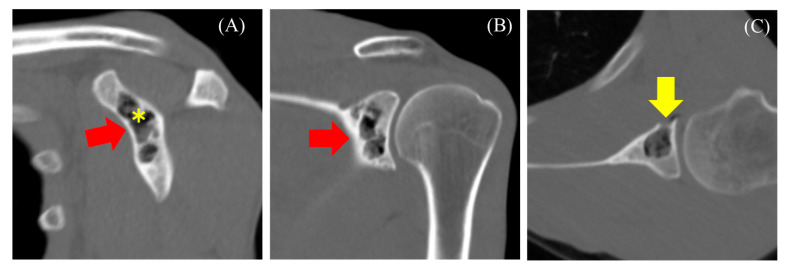
Computed tomography reveals a lobulated translucent image with a sclerotic margin and scattered air density within the lesion ((**A**–**C**) red arrow). The asterisk indicates the location where CT value was measured. Bone defects in certain areas suggest communication with the joint ((**C**), yellow arrow).

MRI revealed an intraosseous tumor with a lobulated morphology consisting of clustered small nodules. The lesion had a low signal compared with that of muscles on T1-weighted images (Figure 2A,D) and a high signal on T2-weighted images (Figure 2C) and fat-suppressed T2-weighted images (Figure 2B,E). The lesion exhibited heterogeneity, with areas of both low signal on T1- and T2-weighted images. No diffusion restriction was observed (Figure 2F,G). Additionally, there was no injury to the rotator cuff or labrum. Based on the MRI findings, an enchondroma or fibrous dysplasia (FD) was suspected, and the patient was referred to our hospital for further treatment, including biopsy and surgery. A plain CT scan was performed to assess the presence or absence of fracture and calcification. CT revealed a lobulated translucent image with a sclerotic margin and scattered air density (−765 Hounsfield unit (HU), Figure 3A asterisk) within the lesion (Figure 3A–C). No internal calcifications or periosteal reactions were observed. The lesion size was 1.5 × 1.2 × 2.7 cm. Additionally, a bone defect in certain areas suggested communication with the joint (C, yellow arrow). Based on these findings, a diagnosis of pneumatocyst was confirmed. The patient was placed under continuous monitoring.

### 2.2. Case 2

**Figure 4:**
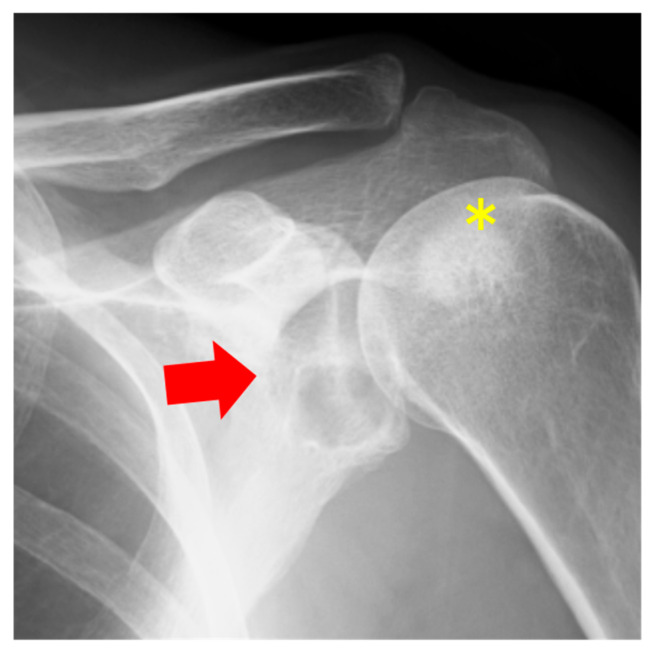
Radiography shows a radiolucent area with a sclerotic margin (red arrow) and sclerotic regions of the humeral head (yellow asterick).

**Figure 5:**
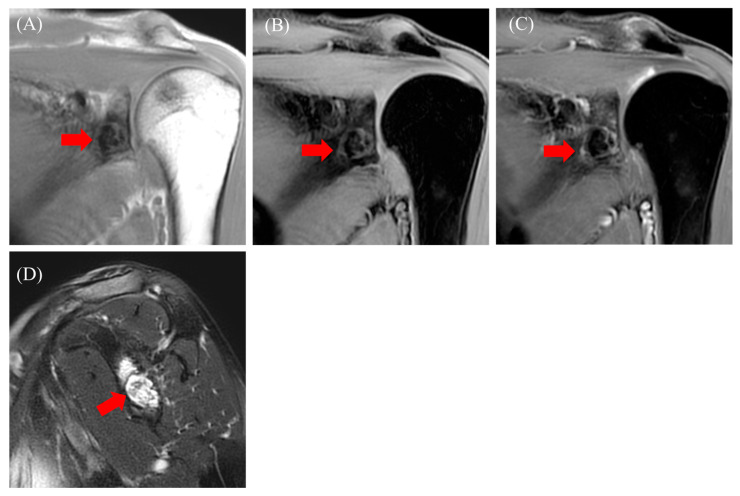
Magnetic resonance imaging: T1-weighted images ((**A**), red arrow), fat-suppressed T1-weighted images ((**B**), red arrow), fat-suppressed T1-weighted images with contrast ((**C**), red arrow), and fat-suppressed T2-weighted images ((**D**), red arrow).

**Figure 6:**
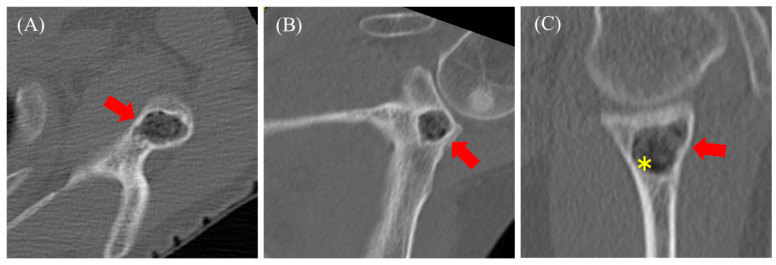
Computed tomography reveals a lobulated translucent image with a sclerotic margin and faint air density within the lesion ((**A**–**C**) red arrow). The asterisk indicates the location where CT value was measured.

The patient was a 57-year-old woman who presented with shoulder pain radiating from the left side of the neck to the shoulder, which began three months ago. She had previously consulted a nearby clinic two months prior. The primary pain site was the trapezius muscle and evator scapulae, with no tenderness in the shoulder joint. The range of motion of the shoulder joint was intact. Additionally, the patient showed no numbness, sensory loss, or muscle weakness in her arm. No history of past illnesses or trauma was recorded. Radiography revealed a radiolucent area with a sclerotic margin in the left scapular glenoid but no narrowing of the joint space (Figure 4). On the other hand, sclerotic regions of the humeral head were observed (Figure 4, asterisk). Suspecting a benign bone tumor of the scapula, an MRI was performed. MRI revealed an intraosseous tumor with a lobulated morphology consisting of clustered small nodules. The lesion had a homogenous low signal compared with that of muscle on T1-weighted (Figure 5A, red arrow) and fat-suppressed T1-weighted images (Figure 5B, red arrow) and a high signal on fat-suppressed T2-weighted images (Figure 5D, red arrow). An enhancement effect was observed in the periphery (Figure 5C, red arrow). MRI showed low signals on both T1- and T2-weighted images without enhancement in the humeral head. Additionally, there was no injury to the rotator cuff or labrum. Based on the MRI findings, a solitary bone cyst (SBC) or FD was suspected, and the patient was referred to our hospital for further treatment, including biopsy and surgery. A plain CT scan was performed to assess the presence or absence of fracture and calcification. CT also revealed a lobulated translucent image with a sclerotic margin and scattered air density (−660 HU, Figure 6C, asterisk) within the lesion (Figure 6A–C). No internal calcifications or periosteal reactions were observed. The lesion size was 1.2 × 1.6 × 1.3 cm. In contrast to Case 1, no continuity was observed between the lesion and the joint. Based on these findings, a diagnosis of a pneumatocyst was confirmed. The patient was placed under continuous monitoring.

## 3. Results and Discussion

A pneumatocyst was first reported in the iliac bone by Ramirez et al. in 1984 [5]. Two systematic reviews on pneumatocysts exist in the literature: one by Oehler et al., which includes cases involving both the spine and extremities [1], and one by Garg et al., which focuses solely on cases involving the spine [2]. The mean age of patients was reportedly in the 50s, while the mean size was 8.67 mm and 11.1 mm, respectively. Oehler et al. suggested that the lesion is more common in male individuals, while Garg et al. reported no significant sex differences, although they focused only on vertebral lesions. The most common sites are the ilium, sacrum, and vertebral bodies [1]. Among the vertebrae, the cervical spine is most frequently affected, particularly C5, followed by C6 and C4 [2]. Rare sites include the clavicle, scapula, humerus, and pubis [1], with only two cases reported in the scapula to date [3,4]. Among the four cases involving the scapula, including the present cases, 75% (3/4) involved male patients, with a mean age of 53 years. Although the size is generally <2 cm, the largest dimension in Case 1 in our report was 2.7 cm. Interestingly, all four cases involved the glenoid. Matsukubo et al. examined the prevalence of intravertebral pneumatocysts (IVPs) in the cervical spine and intradiscal vacuum (IDV) among 500 consecutive patients. The prevalence of IVP and IDV increased with age, being approximately 8% and 6% among individuals in their 40s or below, 30% and 25% among those in their 50s, 49% and 48% among those in their 60s, 55% and 57% among those in their 70s, and 60% and 57% among those in their 80s, respectively [6].

The detailed mechanism of pneumatocysts is currently unknown. Reports indicate that it is observed in 22% cases with adjacent joint or intervertebral disk degeneration, suggesting that these factors may be involved in its formation. Additionally, communication with the joint was observed in approximately 20% cases, as well as in case 1 [1]. One hypothesis is that gas migration occurs from the joints to the bones (approximately 90% nitrogen, oxygen, and carbon dioxide) since pneumatocysts are found near joints [7,8]. Changes in intra-articular pressure can transform these molecules from a liquid to a gaseous state. The intensity and duration of these pressure changes may exceed the resorption capacities of the gases, allowing them to migrate from their initial intra-articular location to adjacent structures, including the bone. Erosive bone lesions exacerbated by chronic microtrauma are sometimes reported in association with vertebral intraosseous pneumocysts [9,10,11]. This mechanism, known as the “vacuum phenomenon”, explains how gas can migrate to the vertebrae. While it may occur physiologically in peripheral joints during intense movements, it is consistently pathological when involving the rachis [7,9,10,12].

The “vacuum phenomenon” may explain the higher occurrence of intraosseous pneumatocysts in older patients and the presence of gas-filled cavities in degenerative osteoarticular lesions, particularly in intervertebral disks. However, it does not fully account for cases in young patients with intraosseous pneumatocysts not associated with degenerative lesions or pneumatocysts located away from such lesions [10,13]. As a second hypothesis, it is believed that mucoid cysts and ganglions present within the bone spontaneously replace gas [5,14]. Based on these findings, Laufer et al. classify pneumatocysts into two types: (1) small-sized pneumatocysts in younger individuals, resulting from the replacement of gas by mucoid cysts or ganglions, and (2) larger-sized pneumatocysts in older individuals due to joint deformation. On the other hand, some believe that cortical erosion due to degeneration, trauma, or congenital factors can explain any type of pathology [15].

Among the two previously reported cases involving the scapula [3,4], the one reported by Kamba et al. showed an osteophyte in the glenoid cavity, indicating the involvement of degenerative changes, while the case reported by Southi et al. did not show any degenerative changes. In both the present cases, there was no joint degeneration or history of trauma. Nevertheless, both Case 1 in our report and the case reported by Southi et al. demonstrated continuity between the scapula and the joint; this suggested the possibility of gas migration from the joint to the bone. Conversely, Case 2 in our report showed no continuity between the scapula and the joint; this indicated that the cystic lesion may have been spontaneously replaced by gas. However, the CT slice width may have influenced the lack of continuity with the joint on the CT scan. Southi et al. recommended 0.625 mm slices to easily detect tiny cortical defects; however, CT was performed with 3 mm slices for our cases, and this may have caused minor defects that were overlooked [3]. In addition, while no continuity was present in Case 2, it is possible that continuity could be observed through long-term follow-up. Therefore, both of the current etiologies may be reasonable.

The most important diagnostic challenge in our cases was the differential diagnosis based on MRI findings. Given the high signal on T2-weighted and fat-suppressed T2-weighted images, the lucent appearance, and marginal sclerosis on plain radiographs in Case 1, we initially considered enchondroma and FD as differential diagnoses. In addition, based on the fat-suppressed T2-weighted images, the differential diagnoses for Case 2 were SBC and FD. The differences in differential diagnoses between the two cases were attributed to the findings on fat-suppressed T2-weighted images. Enchondroma, which was suspected in Case 1, typically shows a high signal on T2- and fat-suppressed T2-weighted images, which reflect cartilage, with low-signal areas reflecting calcifications. The enhancement is limited to the peripheral and septal regions [16]. On the other hand, SBC, which was suspected in Case 2, typically shows homogenous high signals on T2- and fat-suppressed T2-weighted images, which indicate the presence of liquid. The enhancement is limited to the peripheral and septal regions [17]. In FD, which was suspected in Cases 1 and 2, T2- and fat-suppressed T2-weighted images show various degrees of enhancement from high to iso enhancement, and the degree of enhancement can vary significantly among cases [18]. One key feature that suggests a pneumatocyst on MRI is the presence of a gas-fluid cavity [3]; however, this finding was not observed in both our cases. Currently, the positivity remains unclear, and further study will be needed. Typically, pneumatocysts show minimal enhancement at the peripheral margins [3], as observed in Case 2. Considering that both enchondromas and FD do not show strong enhancement, their utility seems to be limited [3,16,18].

Regarding bone tumors originating from the scapula, the scapular body is the most common site, while the glenoid is relatively rare [19]. In benign tumors, the frequency is higher for osteochondromas, followed by osteoid osteomas and cases of enchondromas. Although rare, cases of FD and SBC have also been reported [20,21,22]. Interestingly, in the scapula, SBC on radiography showed marginal sclerosis and a septa-like soup bubble appearance, while MRI displayed a homogeneous high signal on T2 with rim enhancement at the periphery and internal septation. Additionally, in the scapula, FD on radiography showed expandable marginal sclerosis with irregularity, and MRI demonstrated a high signal on short TI inversion recovery. Unfortunately, imaging findings of scapular enchondroma are unavailable; however, it is difficult to differentiate between pneumocystis, FD, and SBC in radiographs and MRI. On the other hand, malignant bone tumors occur in the order of metastases, chondrosarcomas, and Ewing sarcomas. Interestingly, in cases involving individuals under 15 years of age, there is no significant difference in the frequency of benign and malignant bone tumors. However, in adults, malignant bone tumors are more common in the scapula. Radiographic findings tend to show sclerosis for benign tumors and osteolysis for malignant tumors [19]. Since pneumocysts are more likely to develop in older individuals, special attention should be given to distinguishing them from malignant bone tumors. From these perspectives, CT is diagnostically valuable for differentiating pneumatocysts from other bone tumors. Gas density on CT images ranges from −580 to −950 HU, and it was −765 HU and −660 HU in Cases 1 and 2, respectively [1]. In both cases, CT was not initially performed under suspicion of a pneumatocyst; rather, it was performed to check for fractures or calcifications that were not evident on radiographs and to prepare for biopsy. However, CT revealed continuity with the joint and showed the presence of air, playing a significant diagnostic role in this case.

Finally, regarding the prognosis and treatment, there are reports that the size of the mass remains unchanged over the years, while there are also reports that it increases for 14 and 16 months [23,24]. In the cases examined by Kitagawa et al. [23], interestingly, new lesions were observed in adjacent vertebrae as the mass increased. In Wilkinson’s cases, spontaneous absorption of lesions in adjacent vertebrae was also observed [24]. On the other hand, there are reports of cases where the gas was replaced by fluid for several months [25]. While biopsies are not often performed, they were performed in certain cases, revealing subchondral cysts surrounded by a fibrous, nonspecific capsule of mesenchymal origin [5,13,14,26]. Similarly to previous cases, biopsies were not performed in both our cases; this highlights a limitation of these cases and the importance of imaging follow-up. Cases 1 and 2 were followed up for 6 months and 1 year, respectively, during which no changes were observed on X-rays. Generally, surveillance is considered unnecessary once a diagnosis is made. However, there are cases where expansion is observed, and it may be appropriate to follow up for about 2–3 years. There have been no reports of malignant transformation to date. Treatment is mostly conservative, but in cases where symptoms are directly or indirectly exacerbated, fracture risk could increase [8,27,28]. The types of surgery have been reported, including surgical excision with bone graft, alcoholization, or scan-guided injection of micro-balls or cement, although these are thought to be limited [1].

## 4. Conclusions

We presented two rare cases of pneumocysts originating in the scapula. While MRI is undoubtedly useful in diagnosing bone tumors, CT offers a clear advantage in detecting gas within the tumors. It is difficult to consider rare conditions such as pneumocyst in the initial differential diagnosis. Although the usefulness of MRI in diagnosing bone tumors is undeniable, it is not suitable for determining the presence of gas or communication with joints. Thus, it is important to select diagnostic modalities carefully, with particular consideration for the advantages of CT.

## Data Availability

No new data were created or analyzed in this study. Data sharing is not applicable to this paper.

## Abbreviations

The following abbreviations are used in this manuscript:

MRI	magnetic resonance imaging
CT	computed tomography
FD	fibrous dysplasia
SBC	solitary bone cyst
